# A novel GCGR/GLP-1R dual-agonist TB001 ameliorates kidney fibrosis via inhibiting PERK-mediated endoplasmic reticulum stress pathway

**DOI:** 10.3389/fimmu.2025.1665860

**Published:** 2025-11-07

**Authors:** Weijie Lai, Linjie Peng, Jianliang Min, Longhui Qiu, Chang Wang, Shuangjin Yu, Qihao Li, Ruobing Li, Xianxing Jiang, Guodong Chen

**Affiliations:** 1 Organ Transplant Center, The First Affiliated Hospital of Sun Yat-sen University, Guangzhou, China; 2 Guangdong Provincial Key Laboratory of Organ Medicine, Guangzhou, China; 3 Guangdong Provincial International Cooperation Base of Science and Technology (Organ Transplantation), Guangzhou, China; 4 The Second Affiliated Hospital of Southern University of Science and Technology, Shenzhen, China; 5 School of Medicine, Jiaying University, Meizhou, China; 6 School of Pharmaceutical Sciences, Sun Yat-sen University, Sun Yat-sen University, Guangzhou, China

**Keywords:** renal fibrosis, GLP-1r, GCGR, EMT, pERK, er stress

## Abstract

**Background:**

Chronic kidney disease (CKD) affects over one million individuals worldwide and remain a critical economic and healthcare burden. Renal fibrosis is the hallmark of CKD. Previous reports showed that GLP-1R agonists could help prevent kidney fibrosis in diabetic patients. In this study, we aimed to determine the efficacy of a novel GLP-1R and GCGR co-agonist, TB001 in the development of renal fibrosis using both *in vitro* and *in vivo* models.

**Methods:**

Unilateral ureteral obstruction (UUO) surgery was performed on adult B6 mice to establish a mouse model of kidney fibrosis. Mice that underwent sham surgery served as the control group. UUO mice were treated with vehicle or TB001 daily post-surgery and were sacrificed at day 14. Tissue samples were collected for immunohistochemistry and kidney mRNA gene expression analysis. Mouse tubular cells (mTECs) stimulated with TGF-β were used to model kidney fibrosis *in vitro*.

**Results:**

Compared with vehicle treatment, TB001 treatment significantly improved renal histopathology and reduced interstitial collagen deposition and macrophage infiltration in obstructed kidneys. Both *in vitro* and *in vivo* data suggested that TB001 treatment significantly inhibited tubular cell epithelial-mesenchymal transition (EMT). Moreover, the obstructed kidneys in the TB001 treatment group showed significantly fewer PERK and p-eIF2α positive cells than compared to those in the vehicle group, indicating that PERK-mediated ER stress may be involved in the protective effect of TB001 on renal fibrosis. These data were corresponding with the vitro results showing that TB001 significantly suppressed the expression of PERK and CHOP and enhanced mitochondrial mass during TGF-β induced EMT.

**Conclusion:**

This study demonstrated that TB001, a novel GCGR/GLP-1R co-agonist, effectively attenuates renal fibrosis in pre-clinical models, potentially through the inhibition of PERK-mediated ER stress in tubular cells.

## Introduction

Chronic kidney disease (CKD) remains a substantial burden to the health care system, with a global prevalence rate of 9.1%, and is associated with increased morbidity and mortality of cardiovascular disease (1, 2). CKD can progress to end stage renal diseases (ESRD) that requires dialysis or renal replacement therapy. According to reports, as many as 1.2 million people worldwide died from CKD in 2017 (1). Multiple risk factors—including diabetes, hypertension, obesity, infectious insults, and autoimmune diseases have contributed to the development of CKD (3). Regardless of the initial etiology, kidney fibrosis is the final histological hallmark of CKD, which is characterized by excessive production and deposition of pathological extracellular matrix proteins in the interstitium, leading to structural damage, impaired renal function, and ultimately ESRD (4). Despite the progress in the management of CKD, few FDA-approved drugs are available for the treatments of renal fibrosis, and challenges remain in the investigation of therapies for renal fibrosis.

Similar to the wound healing response, kidney fibrogenesis is a dynamic and complex process. The key events of renal interstitial fibrogenesis include peritubular infiltration of inflammatory cells that secrete multiple pro-fibrotic stimuli such as TGF-β and IL-4, tubular epithelial cell apoptosis, epithelial-mesenchymal transition (EMT), and activation and expansion of myofibroblasts (5). Mounting evidence from recent studies identifies endoplasmic reticulum (ER) stress as both a key driver of renal fibrosis and a promising therapeutic target (6).

Glucagon (GCG) and glucagon-like peptide 1 (GLP-1) are peptides encoded by the preproglucagon gene and play critical roles in glucose metabolism (7). Therapies based on GCG and GLP-1 have been approved for the treatment of diabetes and obesity. Glucagon receptors (GCGR) and GLP-1 receptors (GLP-1R) are abundantly expressed in various kidney cell types. Previous studies in animal models and humans have demonstrated that GLP-1R agonists exert protective effects in diabetic nephropathy and acute kidney injury by reducing inflammation, oxidative stress, and lipid accumulation (8).

However, studies on the effects of GCGR or GLP-1R agonists on renal fibrosis and ER are still limited. Moreover, recent studies suggest that dual GCGR/GLP-1R agonists may have stronger pharmacological effects than single agonists (9).

While GCGR/GLP-1R dual agonists like cotadutide and survodutide have advanced into clinical studies, the investigative focus remains largely on diabetes, obesity, and liver fibrosis (10–12). Evidence for renal protection is limited to a single phase 2b trial showing cotadutide improved renal function in patients with Type 2 Diabetes and CKD (13).

TB001, a novel dual agonist of GLP-1R and GCGR synthesized by the School of Pharmaceutical Sciences at Sun Yat-sen University, distinguishes itself from conventional dual agonists by exhibiting higher affinity for GCGR. Additionally, TB001 demonstrates rapid absorption, a short half-life, and negligible drug accumulation *in vivo*. Whether dual GCGR/GLP-1R agonists confer protection against renal fibrosis remains unclear. In our previous studies, we found that TB001 exhibited significant anti-fibrotic effects in liver fibrosis models and renal transplantation models (14, 15).

In this study, we aimed to investigate whether TB001, a novel dual GCGR/GLP-1R agonist, ameliorates renal fibrosis by inhibiting the PERK-mediated endoplasmic reticulum stress pathway, using well-established *in vivo* and *in vitro* models.

## Methods

### Animals, mouse surgery, and treatments

Six to eight weeks old B6 (H2b) male mice were purchased from The Animal Center of Sun Yat-sen University. All mice were used according to protocols approved by the Internal Animal Care and Use Committee of Sun Yat-sen University. The surgical procedure for mouse unilateral ureter obstruction (UUO) was modified based on the method as previously described (16). Briefly, the mouse abdominal skin was cut with surgical scissors. The intestines were moved to the right side of mice with a cotton swap. The left ureter was ligated with an 8-0 suture. The abdomal incision was then closed. For the mice undergone sham operation, mouse abdomen was opened, the intestines were moved out and placed back, and then the abdomen was closed. The mice undergone UUO surgery were treated with vehicle (saline, 200ul) or with TB-001 (80ug/kg) via intraperitoneal injection for 14 days post-surgery. Mice were randomly divided into three groups (1): Sham group (n = 4) (2); Vehicle group (n = 8) (3); TB001 group (n = 8).

### Histology

Histology and immunohistochemical (IHC) staining of kidney sections were performed as described previously. Histologic sections (4μm) were stained with Trichrome Masson and antibodies including anti-E-cad, anti-α-SMA, anti-PERK, and anti-p-eIF2α. The slides were evaluated blindly by two pathologists for the assessment of morphologic characteristics.

### *In vitro* cell culture

Renal tubular epithelial cells (mTECs) were initially isolated from mouse kidneys. The mTECs were then cultured in Renal Epithelial Cell Growth Medium 2 with supplements at 37°C with 5% CO2. For the *in vitro* experiments, mTECs were seeded in 6-well or 24-well plates overnight, and then exposed to 10 ng/ml TGF-β1 or 10 umol/L TB001 for 4hrs or 48hrs.

### Protein extraction and western blot

The mTECs treated with TGF-β1 and/or TB001 were harvested at 48 hours post-treatment. Cells were lysed on ice with lysis buffer containing proteinase inhibitors and phosphatase inhibitors and proteins were extracted according to the reported protocol. Protein concentration was quantified by BCA assay. The proteins were then mixed with loading buffer and boiled. For the western blot experiment, proteins were resolved on 10% SDS-PAGE in running buffer, transferred to polyvinylidene difluoride (PVDF) membranes, blocked with BSA buffer, and incubated with primary antibodies including anti-PERK [(C33E10) Rabbit mAb, Cell Signaling Technology, #3192, 1:1000], anti-p-PERK [(Thr980) (16F8) Rabbit mAb, Cell Signaling Technology, #3179, 1:1000], anti-CHOP [(L63F7) Mouse mAb, Cell Signaling Technology, #2895, 1:1000], and anti-β-actin (anti-β-actin MouseMonoclonal Antibody, ProteinFind^®^, HC201, 1:1000) at 4-Celsius degree overnight. Blots were washed and incubated with the secondary antibody. After washing three times with PBST, signals were detected by chemiluminescence and visualized by ChemiScope 3300 Mini Imaging System.

### Real-time polymerase chain reaction

Total RNA was extracted from kidneys with Trizol reagent according to the manufacturer’s instructions (Thermo Fisher Scientific). One microgram of RNA was used to perform the reverse transcriptase reaction using the qScript cDNA Synthesis kit (Quanta Biosciences, Gaithersburg, MD). The real-time qPCR was run on a Bio-Rad IQ2 PCR machine, and each PCR mixture contained 40 ng of cDNA template and 10 nM primers in 15 μl of SYBR green reaction mix (Bio-Rad). The expression values of each gene were normalized to those that were obtained with the control GAPDH. Changes in gene expression levels were calculated using the 2^−ΔΔCt^ methods. Primer sequences used were showed in **Table 1**.

**Table 1:** Primer sequences of qPCR for mouse.

Target gene	Forward primer (5′-3′)	Reverse primer (5′-3′)
COL1a	ATGTTCAGCTTTGTGGACCTC	CAGAAAGCACAGCACTCGC
XBP1s	CTGAGTCCGAATCAGGTGCAG	GTCCATGGGAAGATGTTCTGG
PERK	AGTCCCTGCTCGAATCTTCCT	TCCCAAGGCAGAACAGATATACC
ATF6	CGGTCCACAGACTCGTGTTC	GCTGTCGCCATATAAGGAAAGG
GAPDH	ACTCCACTCACGGCAAATTC	TCTCCATGGTGGTGAAGACA

### RNAseq analysis

The mTECs treated with or without TGF-β1 (10 ng/ml) were collected at 48 hours post-treatment. The RNA of these samples (n=3/group) was extracted and underwent RNAseq analysis. The RNA integrity was evaluated using the RNA Nano 6000 Assay Kit of the Bioanalyzer 2100 system (Agilent Technologies, CA, USA). A total amount of 1 μg RNA of each sample was used as input material for the RNA sample preparations. Sequencing libraries were generated using NEBNext^®^ UltraTM RNA Library Prep Kit for Illumina^®^ (NEB, USA) following manufacturer’s instruction and index codes were added to attribute sequences to each sample. The clustering of the index-coded samples was performed on a cBot ClusterGeneration System using TruSeq PE Cluster Kit v3-cBot-HS (Illumia) according to the manufacturer’s instructions. After cluster generation, the library preparations were sequenced on an Illumina Novaseq platform and 150 bp paired-end reads were generated. The differentially expressed genes (DEG) analysis was performed using the edgeR package (17) and setting a cutoff CPM of more than 0.4 and an FDR of less than 5%. We used clusterProfiler R package to test thestatistical enrichment of differential expression genes in KEGG pathways (18).

### Statistical analysis

The GraphPad Prism (GraphPad Software, Inc., San Diego, CA) was used for data analysis in this study. Statistical significance between two groups was determined by the Wilcoxon nonparametric tests or by a paired or unpaired t-test. The data representing more than two groups were analyzed with one-way ANOVA. P<0.05 was considered to represent a statistically significant difference. Error bars throughout indicate standard error of the mean (SEM).

## Results

### TB001 significantly attenuated renal fibrosis in mouse models of unilateral ureter obstruction

**Figure 1:**
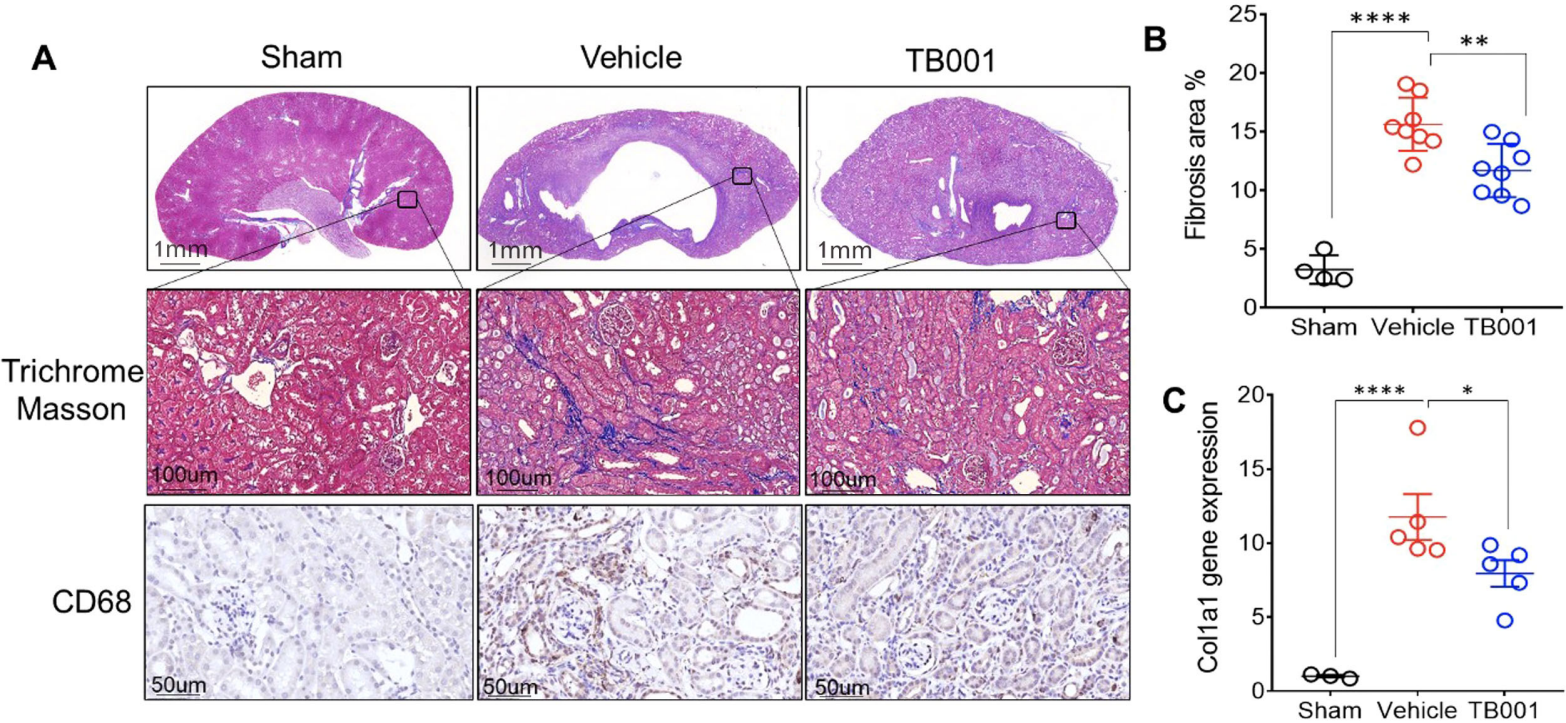
TB-001 treatment ameliorated renal fibrosis in mouse models of unilateral ureter obstruction (UUO). Mouse models of UUO were used to evaluate the efficacy of TB-001 in preventing renal fibrosis. Mice undergone sham surgery (n=4) was used as the control. The mice undergone UUO surgery were treated with vehicle (saline, 200ul; n=8) or with TB-001 (80ug/kg; n=8) via intraperitoneal injection for 14 days post-surgery. The UUO kidneys were harvested on day 14 post-surgery for immunohistochemistry staining. **(A)** The representative Trichrome Masson staining and CD68 staining slides of each group were shown. The fibrosis area of the kidneys **(B)** was calculated based on the histological slides of Masson’s trichrome staining using the Image J software. **(C)** Gene expression of Collagen Type I α1 Chain (Col1a1), a gene that encodes collagen type 1 was analyzed using qPCR method with the indicated kidney tissues. *p<0.05, **p<0.01, ****p<0.0001, one-way ANOVA.

The mouse model of unilateral ureter obstruction (UUO) was used to determine the efficacy of the dual GCGR/GLP-1R agonist, TB001 in the protection of renal fibrosis. UUO was performed in 8- to 12-week-old mice. Kidney morphology and fibrosis were studied 14 days post-obstruction. As shown in **Figure 1**, obstructed kidneys displayed injured tubules with areas of infiltrating cells. Masson’s trichrome staining revealed extensive collagen deposition. TB001 treatment significantly improved the kidney histological morphology and decreased collagen deposition. In the TB001 treatment group, the mRNA of the collagen gene Col1a also decreased compared to the untreated group. Moreover, IHC staining with CD68 showed that obstructive kidneys treated with TB001 showed less macrophage infiltration, indicating attenuated inflammation.

### TB001 inhibited tubular cell epithelial-mesenchymal transition *in vivo* and *in vitro*

**Figure 2:**
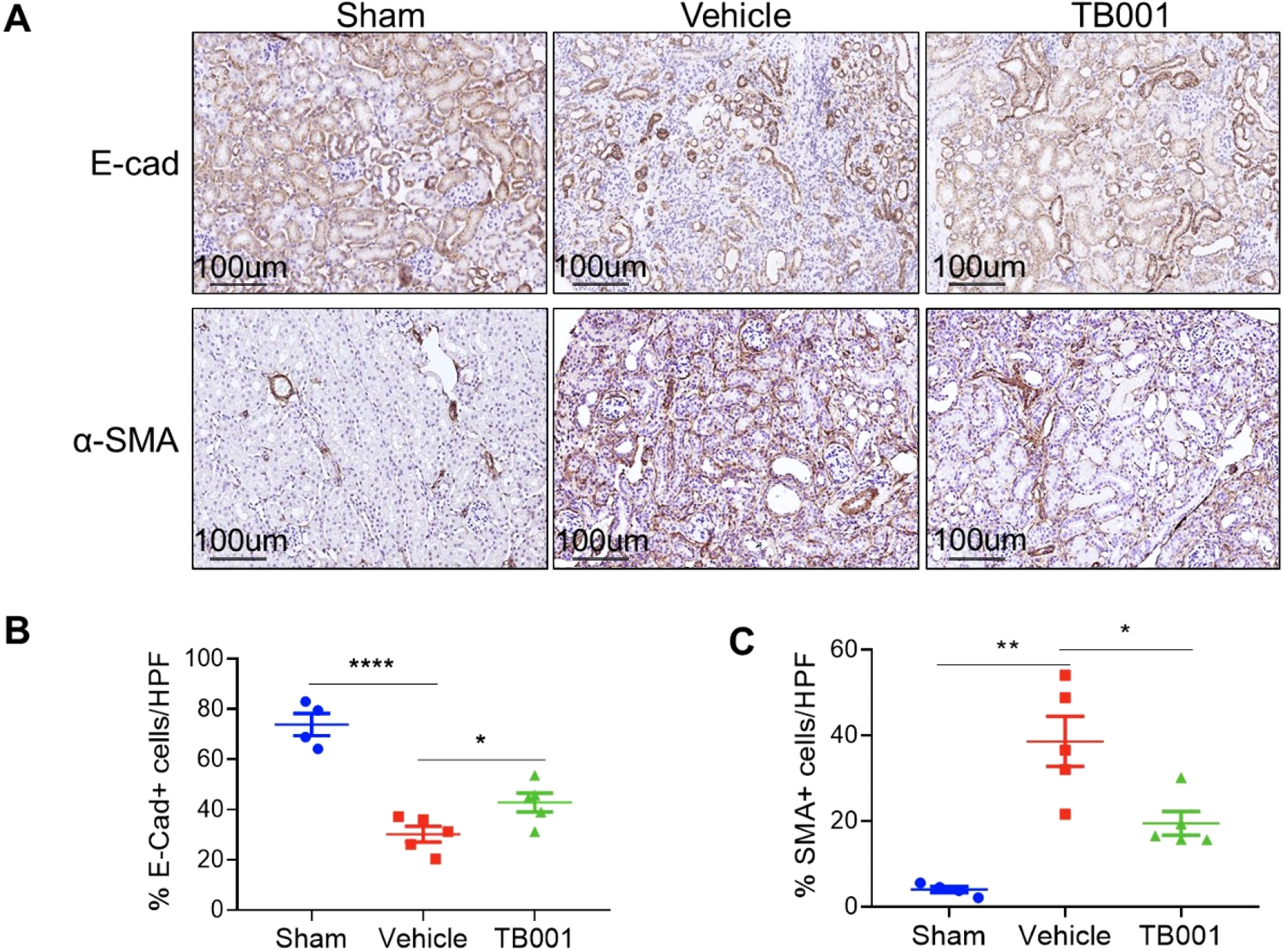
TB-001 treatment inhibited tubular epithelial-mesenchymal transition in the mouse model of UUO. **(A)** Representative IHC images of E-cad and α-SMA staining were shown. **(B)** The percentages of E-cad+ cells and α-SMA+ cells were calculated using the Image J software. *p<0.05, **p<0.01, ****p<0.0001, one-way ANOVA.

Tubular EMT is a critical process of renal fibrosis. To determine whether TB001 plays a role in altering the EMT process, IHC staining for E-cad and α-SMA was performed on kidneys treated with or without TB001. As shown in **Figure 2**, the majority of the normal kidney tubules in the sham operation group were positive for E-cad, and the expression of E-cad in the obstructed kidneys was significantly reduced. However, the TB001 treatment significantly preserved more normal tubules than the vehicle treatment group. It is well known that α-SMA is a well-established marker of fibroblast activation. In kidneys from the sham group, α-SMA expression was mainly confined to blood vessels. In obstructed kidneys, α-SMA in the tubules and tubulointerstitium was significantly up-regulated, and TB001 treatment significantly reduced the frequency of α-SMA positive cells. These results indicate that TB001 suppresses EMT during kidney fibrogenesis.

**Figure 3:**
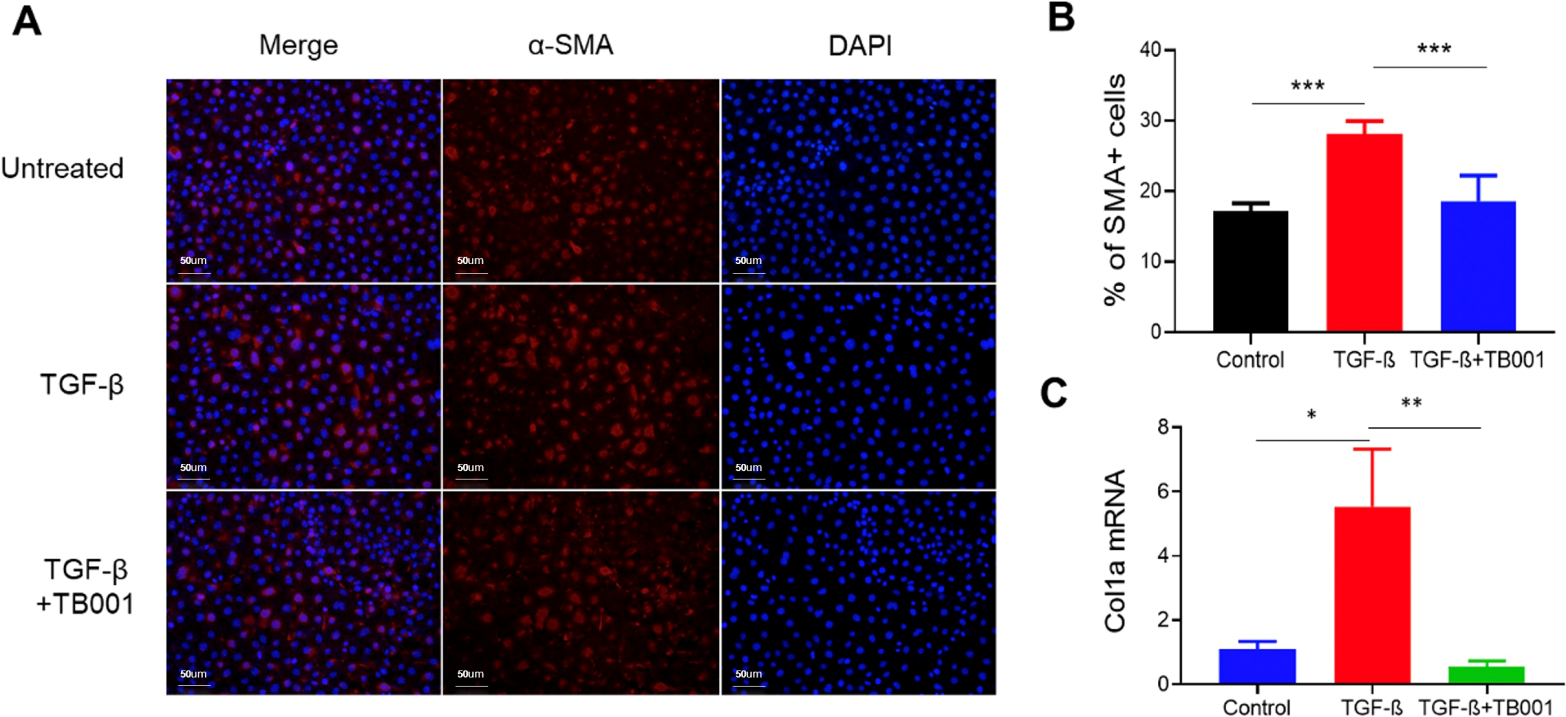
TB-001 treatment inhibited TGF-β induced tubular cell epithelial-mesenchymal transition *in vitro*. **(A)** Representative IF images of α-SMA staining were shown. **(B)** the percentages of α-SMA + cells were calculated. **(C)** Gene expression of Collagen Type I α1 Chain (Col1α1), a gene that encodes collagen type 1 was analyzed using qPCR method. *p<0.05, **p<0.01, ***p<0.001, one-way ANOVA.

To verify this phenomenon *in vitro*, mouse tubular epithelial cells were cultured *in vitro*. EMT process were induced with TGF-β. As shown in **Figure 3**, the addition of TB001 to the culture system significantly inhibited fibroblast activation induced by TGF-β, as indicated by α-SMA staining and col1a genes.

### TB001 suppressed PERK-mediated ER stress pathway during TGF-β induced EMT

**Figure 4:**
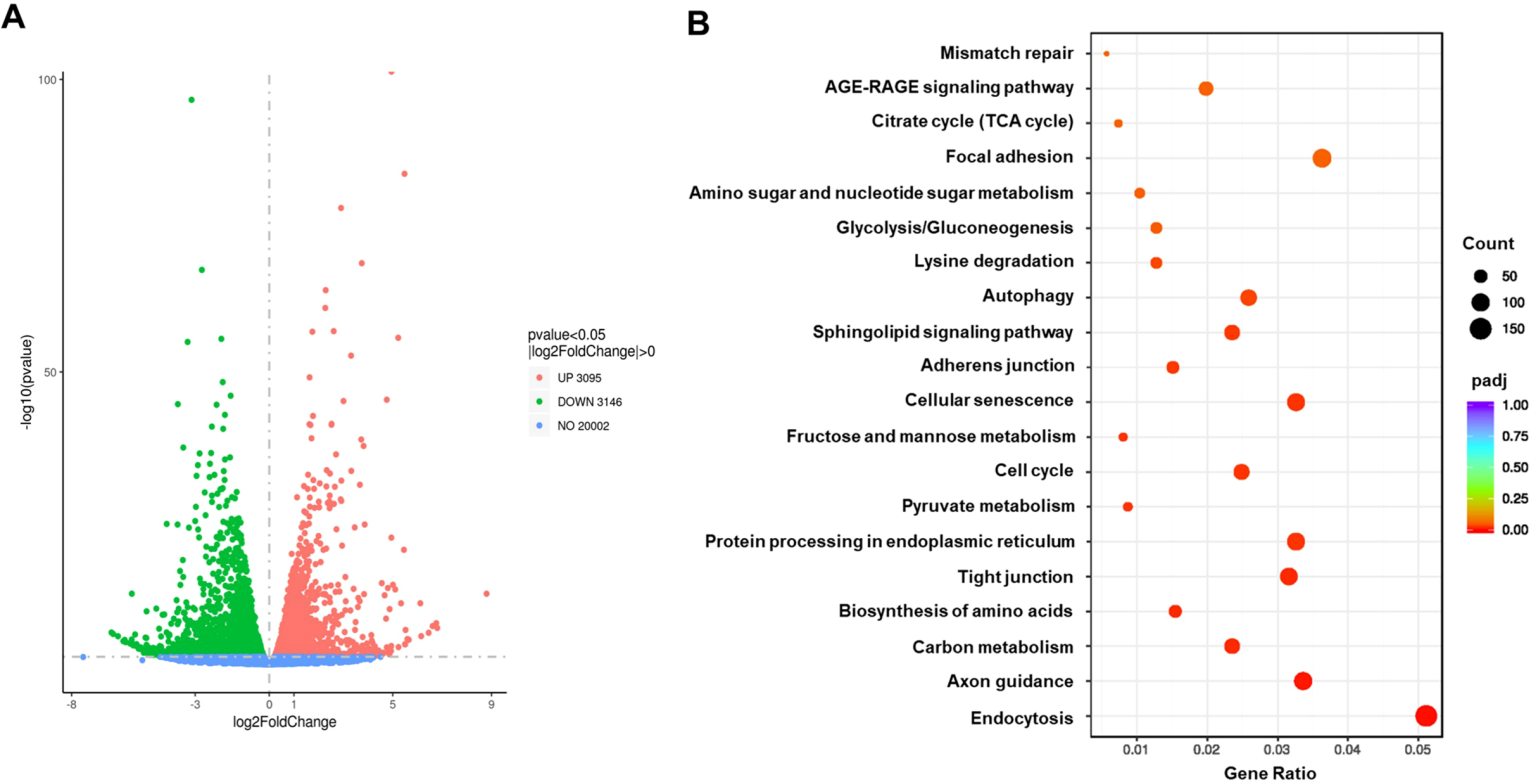
RNAseq analysis of renal tubular cells treated with or without TGF-β. The mTECs treated with or without TGF-β1 (10 ng/ml) were collected at 48 hours post-treatment. The RNA of these samples (n=3/group) was extracted and underwent RNAseq analysis. **(A)** Volcano plot of the differentiated genes. **(B)** KEGG pathway enrichment analysis of differentially expressed genes showed that signaling pathways regulating glucose metabolism and ER stress were both involved in TGF-β induced tubular cell epithelial-mesenchymal transition *in vitro*.

To determine the transcriptome profiles of tubular cells stimulated by TGF-β, we performed the RNAseq analysis of mTECs stimulated with or without TGF-β1 (10 ng/ml) for 48 hours. Over 8000 genes were differentially regulated (**Figure 4A**). The KEGG pathway enrichment analysis of these differentially expressed genes showed that signaling pathways regulating glucose metabolism and ER stress were the top pathways altered in tubular cells after TGF-β stimulation (**Figure 4B**).

**Figure 5:**
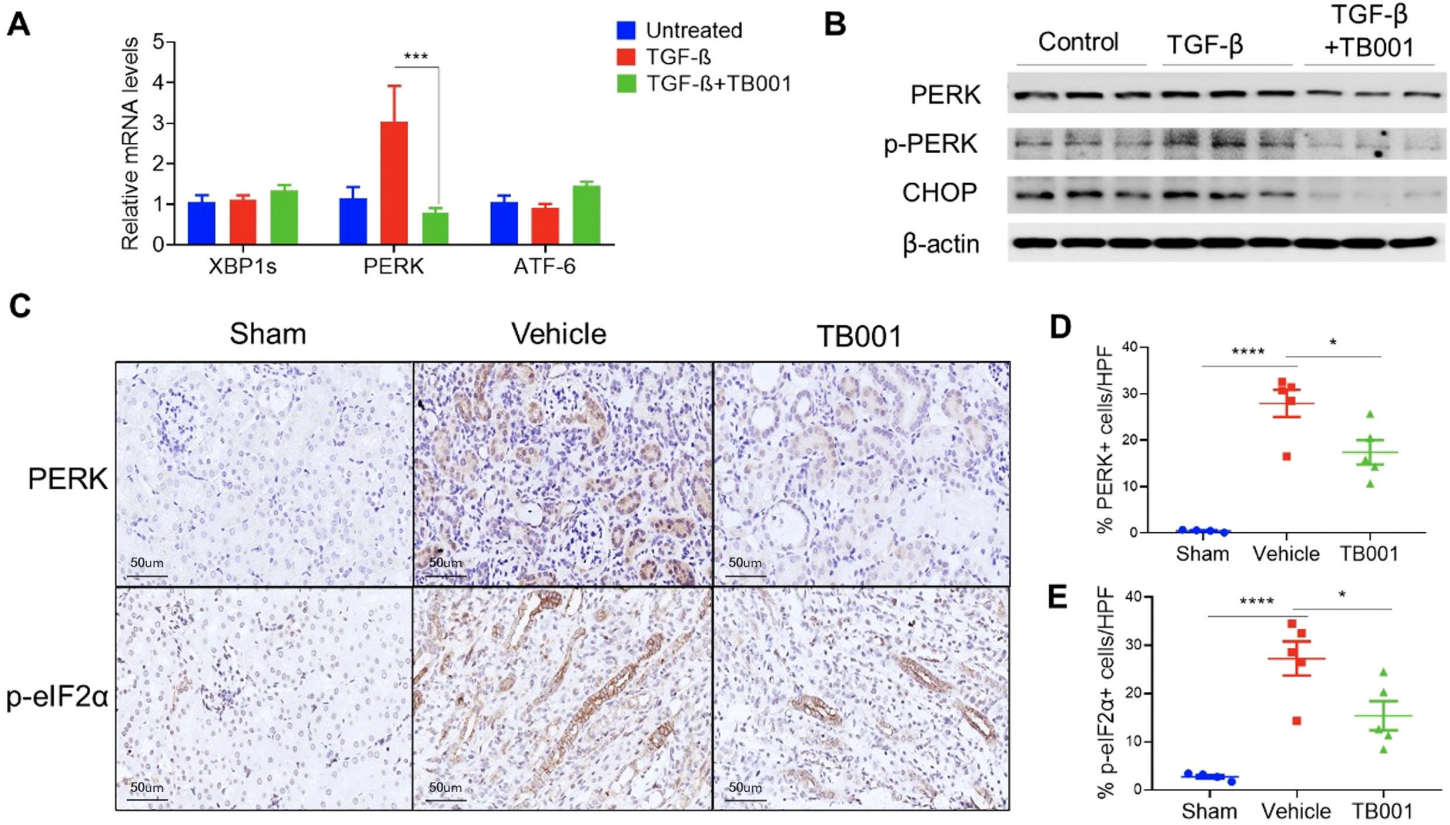
TB-001 treatment decreased PERK-mediated ER stress during EMT *in vitro* and *in vivo*. **(A)** qPCR analysis of ER stress genes including XBP1s, PERK, and ATF-6; **(B)** Western blot analysis of PERK and p-PERK, and CHOP; **(C)** IHC images of PERK and p- eIF2α; **(D, E)** The percentages of PERK and p-eIF2α positive cells. *p<0.05, ***p<0.001, ****p<0.0001, one-way ANOVA.

It has been reported that ER stress pathway plays a critical role in kidney injury and renal fibrosis (19, 20). PERK and CHOP are key mediators of ER stress and contribute to tubular epithelial cell injury and fibroblast activation (21, 22). To investigate whether ER stress is regulated by GCGR/GLP-1R signaling, ER stress genes including XBP1s, PERK, and ATF-6, were analyzed by qPCR (**Figure 5**). The results showed that PERK expression was significantly upregulated in tubular cells under the induction of TGF-β, and was significantly inhibited after TB001 was added. Western blot analysis further demonstrated that TB001 reduced the protein levels of PERK, phosphorylated PERK (p-PERK), and CHOP during TGF-β-induced EMT.

To further validate the *in vitro* findings, PERK and p-eIF2α IHC staining were performed in obstructed kidneys with or without TB001 treatment. As shown in **Figure 5**, PERK and p-eIF2α were mainly localized in injured tubular cells. Compared with normal kidneys in the sham operation group, obstructed kidneys showed significantly increased PERK and p-eIF2α positive tubular cells. However, TB001 treatment significantly reduced the expression of PERK and p-eIF2α in obstructed kidneys. We attempted to perform p-PERK, eIF2α, and CHOP staining in kidney samples but failed due to massive unspecific staining. In summary, these data suggested that TB001 treatment inhibited PERK-mediated ER stress during EMT in the *in vitro* and *in vivo* fibrosis models.

### TB001 improved mitochondrial morphology

**Figure 6:**
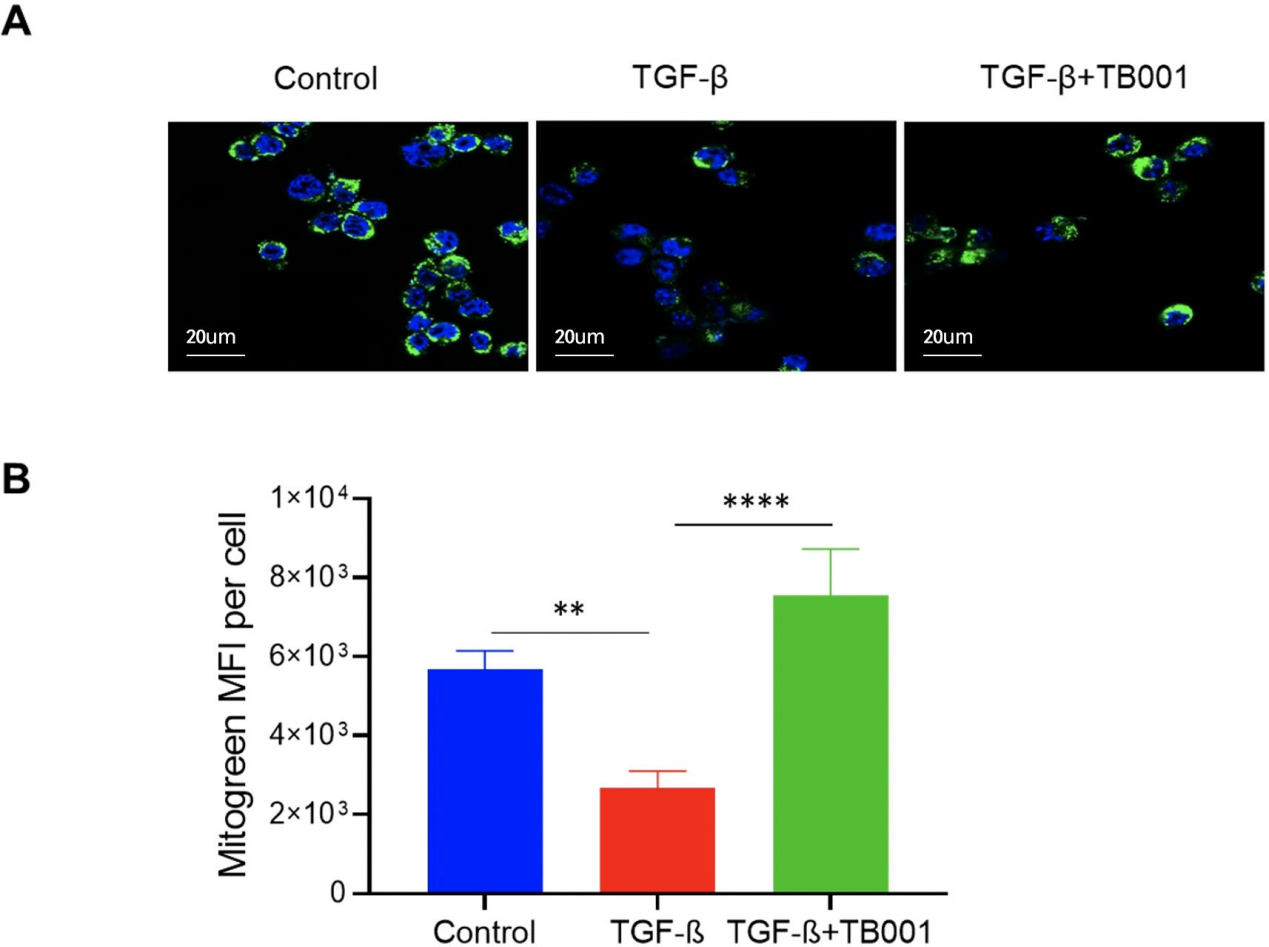
TB-001 treatment improved mitochondrial mass during TGF-β induced EMT. The mTECs were treated with or without TGF-β1 (10 ng/ml), TB001 (umol/L) for 4 hours. The Mitochondria were stained using the Mitogreen (green) and the nuclei were stained using Hoechst (blue). Reprehensive images of cells treated with or without TGF-β1 or TB001 detected by confocal microscopy **(A)** and the Mitogreen intensity **(B)** were shown. **p<0.01, ****p<0.0001, one-way ANOVA.

It has been proven that ER stress pathway closely links to mitochondrial function. Mitochondrial dysregulation secondary to ER Stress has been implicated in various kidney diseases (23). To further determine whether TB001 has effects on the mitochondrial morphology of renal tubular cells, the Mitogreen staining was performed in the *in vitro* cell system. The data showed that TGF-β induction decreased the mitochondrial mass of cultured tubular cells, and this effect was reversed by the addition of TB001 (**Figure 6**), suggesting that TB001 treatment may play a role in the mitochondrial metabolism.

## Discussion

In this study, our results demonstrated that dual GLP-1R and GCGR agonist TB001 could significantly ameliorate renal fibrosis in UUO mouse models and *in vitro* cell culture systems. Also, we determined that PERK-mediated ER stress pathway was involved in the process of renal fibrosis, and TB001 treatment could suppress this pathway and ameliorate chronic kidney injury.

GCGR and GLP-1R agonists have been shown to have protective effects on prevention or conciliation of kidney injury in various disease models. In human settings, most studies were conducted in patients with diabetic kidney disease (DKD), a leading cause of ESRD worldwide (24, 25). Current data from clinical trials suggest that the protective effect of GLP-1R agonists (such as Liraglutide, Semaglutide, Dulaglutide, and Lixisenatide) on the kidneys is mainly driven by the reduction of albuminuria (24). Several experimental studies have also shown that GLP-1R and GCGR agonists may exert their renal protective effects through mechanisms independent of their glucose-lowing effects (26–29). As demonstrated by Tuttle KR, GLP-1 receptor agonists may ameliorate renal fibrosis by reducing inflammation via a mechanism independent of glucose-lowering (26, 27). Peter Boor et al. found that the GLP-1 receptor may attenuate renal tubulointerstitial injury by mitigating immune-mediated accumulation of macrophages and T cells in the kidney (28). According to the reports, GLP-1R agonists could inhibit nicotinamide adenine dinucleotide phosphate (NADPH) oxidase by regulating cAMP-PKA signaling, thereby preventing glomerular and tubular oxidative stress (30, 31). Moreover, studies have shown that GLP-1R agonists may downregulate NF-κB activation in podocytes, glomerular endothelial cells, monocyte/macrophages, and mesangial cells, thereby inhibiting tge inflammatory response in diabetes models (32–35). In non-diabetic settings, Li et al. showed that liraglutide could ameliorate UUO-induced tubulointerstitial fibrosis by inhibiting TGF-β and its downstream signaling pathways including Smad3 and ERK1/2, thereby suppressing renal tubular EMT (36). Similar to this study, our data showed that the novel GCGR/GLP-1R dual agonists TB001 could ameliorate renal fibrosis by downregulating tubular cell EMT (**Figures 1**–**3**). Whether dual agonists of GCGR/GLP-1R have a superior effect in comparison to single agonists of GLP-1R remains to be determined.

The endoplasmic reticulum is essential for many cell functions, including protein synthesis, folding, modification, and transportation. The disruption of these functions by extracellular or intercellular stimuli can affect proper protein folding and result in ER stress. The ER is governed by three ER stress sensors, including PERK, IRE1α, and ATF-6. The PERK pathway is activated by autophosphorylation, which then phosphorylates eIF2α and promotes the expression of pro-apoptotic proteins including CHOP. Deficiency of CHOP can ameliorate renal ischemia-reperfusion injury in mice, and prevents UUO-induced renal fibrosis by attenuating fibrotic signals derived from HMGB1/TLR4/NF-κB/IL-1β signaling (21, 37). Moreover, it is reported that TGF-β interests with ER stress pathway to promote fibrogenesis in liver fibrosis models (38). Currently, whether ER stress pathway plays a role in the renal protection effect of GCGR/GLP-1R agonists remains unclear. Our data support that the GCGR/GLP-1R agonist TB001 could inhibit UUO induced activation of PERK-eIF2α-CHOP signaling in tubular cells (**Figures 4**, **5**). Whether IRE1α or ATF-6 pathway plays a role in the protective effect of TB001 has not been investigated in this study.

Compelling evidence show that ER stress signalings closely cross-talk with mitochondrial pathways in various cell activities including cell death (23, 39), and that ER hyperplasia or ER stress can affect mitochondrial biogenesis and mitochondrial function (40–42). It is reported that, in rat CKD models, the GLP-1R agonist Liraglutide could improve the mitochondrial function of renal cells by activating the SIRT1/AMPK/PGC1α pathway, and reduce lipid accumulation in the kidney (43). In this study, we also observed that TB001 treatment could improve the mitochondrial mass of renal tubular cells stimulated with TGF-β (**Figure 6**).

In this study, we primarily focused on the effect of TB001 on renal tubular cells during kidney fibrogenesis. However, some other mechanisms may also be involved. For example, capillary endothelial-mesenchymal transition (EndoMT) is also a critical factor that contributes to the fibrogenesis, and Sitagliptin and Liraglutide have been shown to ameliorate the EndoMT process of renal fibrosis by inhibiting TGF-β1 (44). Whether TB001 has an effect on reversing EndoMT remains to be investigated.

It should be noted that this study has several limitations. First, due to the impact of COVID-19 pandemic, experiments addressing the require factors of the renal protective effect of TB001 were interrupted, and further mechanisms underlying the link between ER stress/mitochondrial and GCGR/GLP-1R remain to be explored. Second, although our study revealed that TB001 could induce the activation of the PERK–eIF2α–CHOP signaling pathway in renal tubular cells, it is still insufficient to establish this pathway as the dominant mechanism underlying the effects of TB001. Third, the evidence for receptor specificity (GCGR/GLP-1R dependence) remains indirect, and future studies are needed to compare TB001 with single agonists targeting GCGR or GLP-1R alone. Moreover, in this study, we did not investigate the systemic effects that could be altered by the TB001 treatment. Moreover, in this study, we did not investigate the systemic effects that could be altered by the TB001 treatment. In addition, other than the kidney cells, various other cell types in liver, pancreas, and blood vessels also expressed GLP-1R and GCGR; it cannot be ruled out that TB001 exerts its effect through these organs or cell types and indirectly prevents renal fibrosis. Despite these limitations, the data reported in this study strongly supports that TB001 treatment can reduce collagen deposition and tubular EMT in obstructed kidneys, concomitant with decreased PERK-eIF2/CHOP signaling.

## Conclusion

In summary, this study demonstrates that TB001, a novel GCGR/GLP-1R co-agonist, can effectively alleviate renal fibrosis in pre-clinical models, likely by attenuating PERK-mediated ER stress and EMT in tubular cells.

## Data Availability

The original contributions presented in the study are included in the article/supplementary material. Further inquiries can be directed to the corresponding author.
